# Analysis of Basic Physical and Chemical Characteristics of Manganese Slag before and after Solidification and Its Feasibility as Highway Slope

**DOI:** 10.3390/ma14195530

**Published:** 2021-09-24

**Authors:** Meng Chen, Jianming Wei, Runhua Zhang, Lipei Jia, Qiqi Yao, Anchao Han

**Affiliations:** 1 College of Civil Engineering, Chongqing Jiaotong University, Chongqing 400074, China; chenmeng@mails.cqjtu.edu.cn (M.C.); 622180111064@mails.cqjtu.edu.cn (L.J.); 622180111019@mails.cqjtu.edu.cn (Q.Y.); hananchao2021@163.com (A.H.); 2 College of Engineering and Physical Sciences, University of New Hampshire, W161 Kingsbury Hall, 33 Academic Way, Durham, NH 03824, USA; runhua.zhang@unh.edu

**Keywords:** manganese slag, physical and chemical properties, engineering characteristics, Monte Carlo simulation, slope stability

## Abstract

Manganese slag is a kind of industrial waste produced by electrolytic production of manganese metal. The traditional method of stacking manganese slag not only causes waste of resources, but also produces environmental pollution. Finding harmless, effective, and economical disposal technology of manganese slag has gradually become a research hotspot and difficulty in the field of electrolytic manganese industry and environmental protection. To verify the feasibility of using manganese slag as roadbed material, the basic physical and chemical properties of manganese slag were analyzed based on X-ray diffraction, X-ray fluorescence spectrum, SEM scanning electron microscope, and particle analysis, the basic engineering characteristics of raw materials of manganese slag and solidified manganese slag mixed with quicklime were analyzed through a compaction test and a *CBR* test. Finally, based on the Monte Carlo method, the stability of a highway slope in the Guizhou Province of China is simulated by the finite element method, considering the spatial variability of manganese slag material strength parameters. The results show that the solidified manganese slag material can be used as highway subgrade material. This study has important reference significance for manganese slag highway construction projects.

## 1. Introduction

Electrolytic manganese metal is an important metallurgical and chemical raw material, which is widely used in metal alloys, electronic devices, power batteries, construction, sewage treatment, pharmaceuticals, and other industries [1,2,3,4]. It is an important basic material and national strategic resource in the national economy [5,6]. Electrolytic manganese slag is a mixture of acid leaching slag, sulfide slag, and anode slag [7]. There are many heavy metals in electrolytic manganese slag. These heavy metals are transformed through the changes in the natural environment, and the adverse effects on the surrounding people’s health and ecological environment are persistent [8,9]. China is a major producer of manganese slag [10]. In China, manganese slag is mainly stored in wet dams. This not only takes up precious land, but also produces excessive ammonia nitrogen, sulfate, and other harmful heavy metal ions [11], causing serious pollution to the surrounding soil [12], and heavy metals, etc., easily penetrate into the soil, groundwater, and surface water, causing serious environmental pollution and safety risks to aquatic ecosystems and agricultural ecosystems [13], and eventually manganese residue enters the human body through the role of the food chain, affecting human health. At the same time, under the influence of wind and sun, some manganese slag is scattered in the air, which pollutes the surrounding atmospheric environment [14]. A large amount of manganese slag has caused serious social and environmental pollution problems in China, such as water pollution, heavy metal pollution, residue accumulation and solid waste pollution [15,16], etc., Geoff et al. [17] found that an old waste dump can cause potential heavy metal pollution to the ecosystem by analyzing the accident of a tailings dam rupture. Ran et al. [18] analyzed the sources, spatial distribution of metal(loid)s, and the risks to public health at an abandoned arsenic mine site. Therefore, exploring the use of industrial waste residue as a subgrade filler has become the focus of scholars in recent years. Electrolytic manganese residue (EMR), red mud (RM), and carbide slag (CS) are three kinds of solid waste that are largely produced and difficult to recycle. At present, the utilization of these three types of waste residues is mainly focused on the preparation of building materials. For example, EMR is used in autoclaved bricks, ecological cement, geopolymer; RM is used to replace clay for non-burned brick, road materials, and cementitious material; while CS can be used as an alternative for limestone in cement production, or xonotlite [19]. At the same time, landfilling or open-air accumulation is the main disposal method of these residues, which causes harm to the soil and groundwater, thereby harming the ecological environment. Recycling and reusing them can effectively protect the environment. Zhang et al. [19] used electrolytic manganese slag, red mud and calcium carbide slag as road materials, and investigated their mechanical properties, durability, strength formation mechanism and environmental behavior. Using manganese slag as subgrade material and reasonably recycling manganese slag materials cannot only solve the problems of manganese slag storage, environmental pollution and land occupation, but it can reduce the production cost and bring considerable ecological and social benefits.

In this study, the basic physical and chemical characteristics of manganese slag were analyzed based on X-ray diffraction, X-ray fluorescence spectrum, SEM scanning electron microscope, and particle analysis test. The basic engineering characteristics of raw materials of manganese slag and solidified manganese slag mixed with quicklime are analyzed through a compaction test and a *CBR* test. Considering the spatial variation in manganese slag material strength parameters, the stability of a highway slope was simulated by the finite element method. This study has important reference significance for highway construction projects using manganese slag.

## 2. Experimental Study

### 2.1. Basic Physical and Chemical Properties of Manganese Slag

#### 2.1.1. X-ray Diffraction Analysis

The principle of XRD is that X-ray irradiates atoms and molecules in the crystal to produce a diffraction phenomenon, and the diffraction law follows the Bragg’s Law:

2dsinθ = nλ(1)

where d is the crystal plane spacing, θ is the diffraction angle, λ is the X-ray wavelength, and n is the integer. Through the Bragg’s Law, we can use the known X-ray to diffract the crystal with an unknown structure, and use the diffraction angle to calculate the crystal plane spacing, and then we can determine the crystal type and structure of the object to be measured. Escalab250xi X-ray photoelectron spectrometer (Thermo Fisher Scientific Company, 81 Wyman Street, Waltham, MA 02454, USA) produced by Thermo Fisher Scientific Company of the United States was used in the X-ray diffraction analysis test. The main technical specifications of the instrument are shown in Table 1.

**Figure 1:**
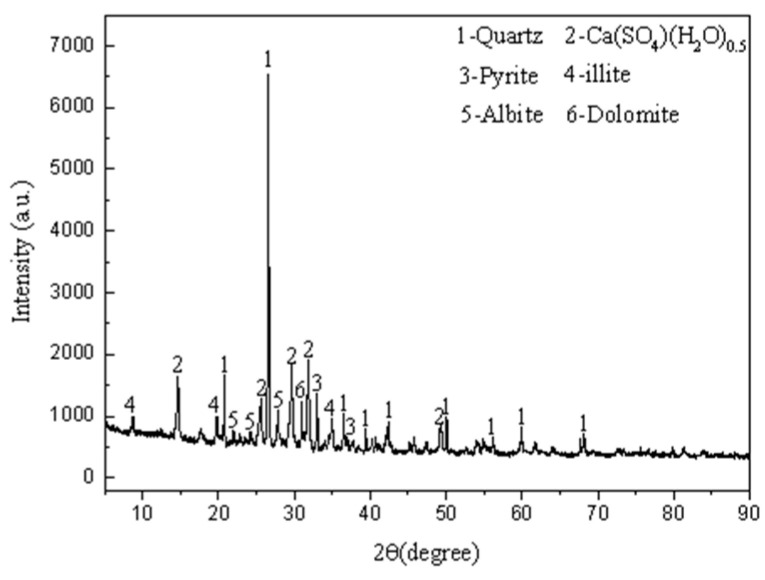
XRD pattern of manganese slag.

The XRD pattern of manganese slag obtained by the XRD test is shown in Figure 1. According to the qualitative analysis of the XRD pattern, there are six types of characteristic peaks in manganese slag, among which quartz is the most prominent, indicating that the crystallinity of quartz in manganese slag is higher.

The second characteristic peak is hemihydrate gypsum (CaSO_4_-(H_2_O)_0.5_), which is mainly formed by the reaction of CaMn(CO_3_)_2_ and CaCO_3_ in manganese carbonate ore with H_2_SO_4_. In addition, a small amount of Pyrite, illite, albite, and dolomite are also found in the manganese slag, which is consistent with the existing literature [20,21,22]. 

#### 2.1.2. XRF Spectrometry Analysis

X-ray fluorescence spectroscopy (XRF) can more accurately analyze the elements qualitatively and quantitatively, and can accurately identify multiple elements. The XRF test instrument used in this study is the ARL Perform X-ray fluorescence spectrometer produced by Thermo Fisher Scientific in the United States. XRF was used to test the element distribution in the manganese slag and calculate the oxide content. The test results are shown in Table 2 and Table 3, respectively.

Table 2 shows the content of elements in manganese slag. It can be seen that the main elements (content > 1%) in manganese slag include O, Si, S, A1, Ca, Fe, Mn, Mg, K, etc., which account for 98.06% of all elements. Among them, the content of O, Si is the highest, accounting for more than 60% of all elements. Table 3 shows the chemical composition of manganese slag. The oxides in manganese slag include SiO_2_, SO_3_, CaO, Al_2_O_3_, Fe_2_O_3_, MnO, MgO, and K_2_O, accounting for 97.59% of all oxides. The content of SiO_2_ and SO_3_ is the highest, accounting for about 60% of the total content, which is in contrast with the XRD analysis, because the main chemical composition of quartz is SiO_2_.

The chemical composition of manganese slag is similar to that of ordinary silicate, mainly clay minerals. The crystal structure and material composition of manganese slag determines its natural form. The fresh manganese slag generally has low water content and presents fine particles; after being stored for a period of time, the manganese slag is eroded by rain and presents the shape of flowable slurry, which is similar to that of general cohesive soil, which is one of the feasible conditions for using manganese slag as subgrade filler.

#### 2.1.3. SEM Scanning Electron Microscope Analysis

**Figure 2:**
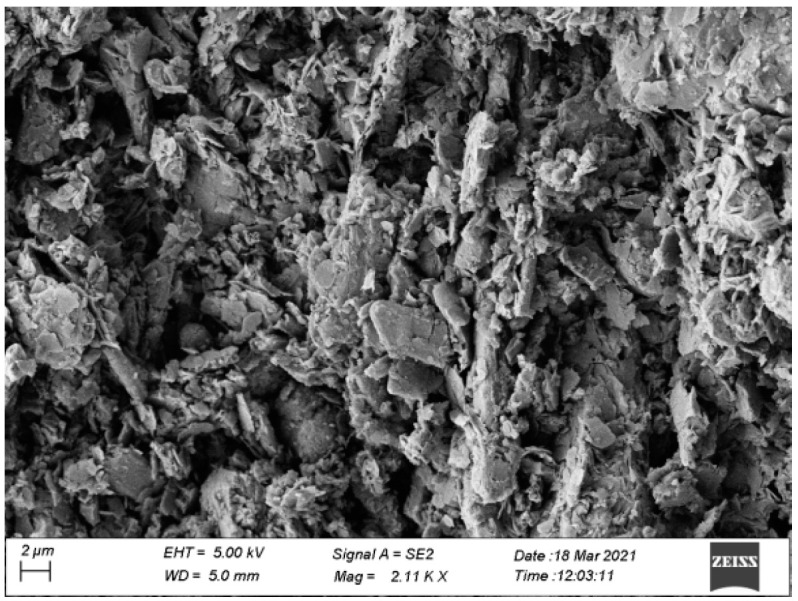
SEM image under 2 μm scale.

**Figure 3:**
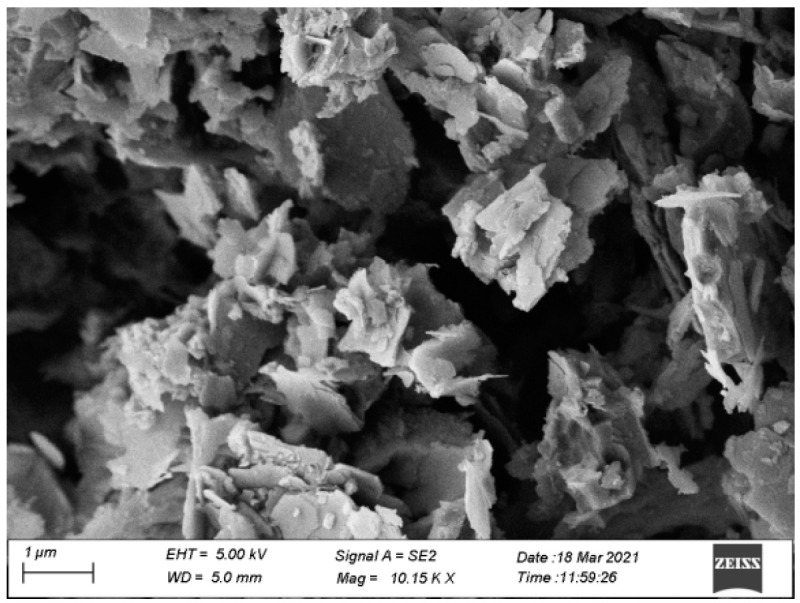
SEM image under 1 μm scale.

In order to clarify the microscopic morphology of natural manganese slag, observe its pore state, aggregate structure characteristics, and other information, scanning electron microscopy tests were carried out on uncured manganese slag samples. The scanning electron microscope images of manganese slag under the scale length of 2 μm and 1 μm are shown in Figure 2 and Figure 3. It can be seen that under the 2 μm scale length, the microscopic morphology of the manganese slag presents massive and strip-shaped crystal particles covered by a large number of spherical flake particles. The scanning electron microscope image under the 1 μm ruler length shows this feature. Combined with the analysis of the XRD and XRF results of the manganese slag, it is judged that the massive and band-like crystal particles in the manganese slag should be hemihydrate gypsum (CaSO_4_-(H_2_O)_0.5_), and many flake and spherical particles attached to its surface may be silicon dioxide crystals (SiO_2_). In addition, the size distribution of manganese slag particles is relatively uneven, there are a lot of pores between the particles, and no obvious cementation is found between the particles. These structural characteristics indicate that the connection between manganese slag particles is not tight, so when manganese slag is used as slope material, solidification treatment should be considered first. 

#### 2.1.4. Particle Analysis Test

**Figure 4:**
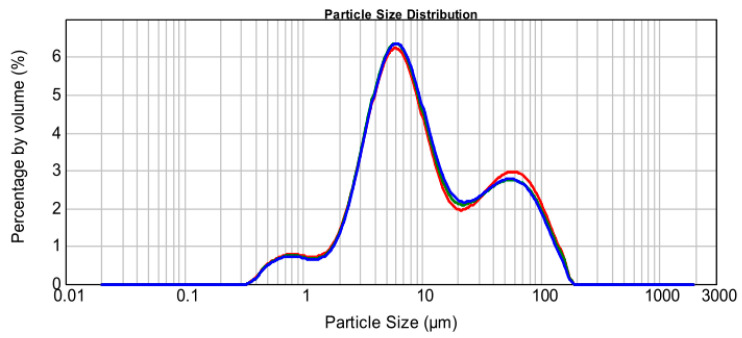
Particle size distribution curve of manganese slag.

By testing the percentage of different particle sizes in the total mass of manganese slag, the particle size distribution curve of manganese slag can be drawn, and the characteristics of particle size distribution can be determined. In this study, Malvern Mastersizer 2000 laser particle size analyzer was used to analyze the particle size distribution of manganese slag. Figure 4 shows the particle size distribution curve of manganese slag, the results of particle size distribution in manganese slag are shown in Table 4. The content of clay in manganese slag is small, accounting for only 7.37% of the total particle size. The proportion of silt and sand is as high as 92.63%, in which silt accounts for 60.20% of the total particle size. 

According to the comprehensive test results, the particle size distribution of manganese slag is similar to that of sandy silt. Its particles are fine and uniform, mainly sand and powder particles, with little clay content, making sand and powder particles relatively large. The voids between the particles are not filled with fine clay particles, forming a so-called “building block” type framework, which makes it difficult to compact. Therefore, from the perspective of particle size distribution, manganese slag is not suitable for direct use as roadbed filler.

### 2.2. Engineering Characteristics before Solidification

#### 2.2.1. Compaction Test

The compaction characteristics of subgrade fillers can be determined through indoor compaction tests. The maximum dry density and optimal moisture content are of great significance for guiding construction, which can be determined by controlling different compaction powers to hammer filler samples with different moisture contents. A standard light compaction test was carried out on the manganese slag sample, the dry density corresponding to different moisture contents after compaction was calculated by the following formula, and the relationship between moisture content and dry density was drawn. The curve is the compaction curve.

(2) $$\rho_{d}=\frac{\rho }{1+0.01\omega }$$

where: $\rho_{d}$, dry density of manganese slag (g/cm^3^); ρ, the wet density of manganese slag (g/cm^3^); ω, Water content (%).

**Figure 5:**
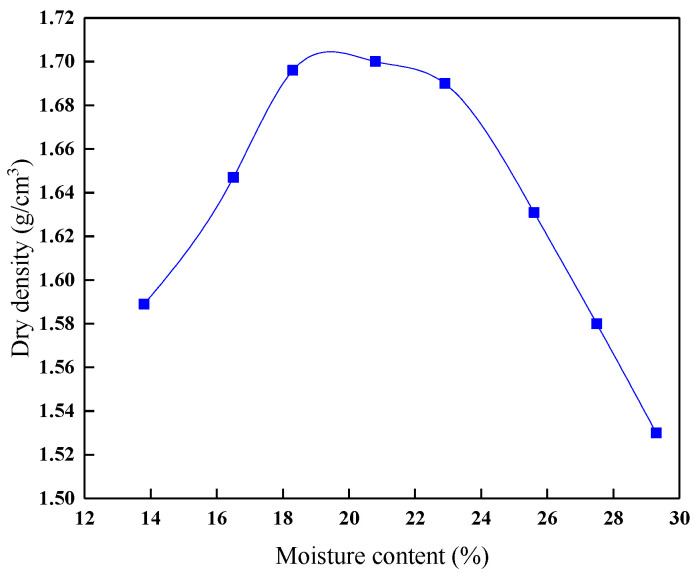
Compaction test curve.

The maximum dry density reflects the densest state that the soil can reach within a certain range of water content under the action of a certain compaction work. According to the test record data in Table 5, the compaction curve is drawn as shown in Figure 5. The compaction curve of manganese slag is similar to that of soil. The dry density first increases and then decreases with the gradual increase in water content. The optimal water content is 20.1%, and the corresponding maximum dry density is 1.71 g/cm^3^. Compared with the natural moisture content of 32.7%, the optimal moisture content of manganese slag is obviously lower than the optimal moisture content. Therefore, when using manganese slag as subgrade filler, how to reduce its moisture content to the optimal moisture content should be considered to achieve the best compaction effect.

#### 2.2.2. *CBR* Test

*CBR* can be used to evaluate the load-bearing properties of materials. The *CBR* value refers to the ratio of the unit pressure to the standard load strength (7 Mpa or 10.5 Mpa) when the penetrator penetrates the sample 2.5 mm or 5 mm when the standard crushed stone is pressed into the same penetration amount, expressed as a percentage. At present, the *CBR* value was used as an important basis for the selection of roadbed fillers. The unit pressure can be calculated based on the dynamometer reading, and the relationship curve between unit pressure and penetration can be drawn, and the unit pressure value when penetration is 2.5 mm and 5 mm can be found on the curve, and the *CBR* value can be calculated according to the following formula:

(3) $$CBR_{2.5}=\frac{P}{7000}\times 100$$

(4) $$CBR_{5.0}=\frac{P}{10500}\times 100$$

where: *P*, unit pressure, *CBR*_2.5_ is the *CBR* value of manganese slag when the penetration is 2.5 mm; *CBR*_5.0_, the *CBR* value of manganese slag when the penetration is 5 mm.

**Figure 6:**
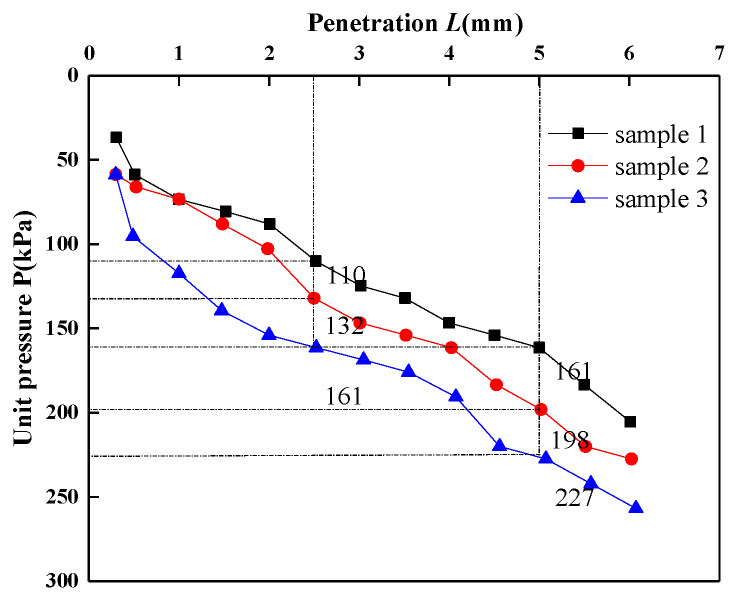
The relationship curve between unit pressure and penetration.

*CBR* tests were carried out on three groups of manganese slag samples, and the relationship curve between unit pressure and penetration is shown in Figure 6. According to the curve, the unit pressure when the penetration is 2.5 mm and 5 mm can be found, the *CBR* value can be calculated. Table 5 shows that the *CBR* value of manganese slag is between 1.5 and 2.2, which does not meet the construction strength standard of highway subgrade; therefore, manganese slag cannot be directly used as subgrade filler. 

In addition, the expansion amount and expansion rate of compacted manganese slag samples were also tested. It was found that the manganese slag had a certain water absorption after compaction, the water absorption amount was between 203 and 238 g, and the expansion rate was between 6.13 and 6.46. Therefore, it was judged that manganese slag had micro expansion.

### 2.3. Engineering Characteristics after Solidification

The *CBR* value of manganese slag is between 1.5 and 2.2, which does not meet the construction strength standard of highway subgrade, and the gap between manganese slag particles is not filled with fine clay particles, which makes it difficult to compact. Therefore, from the point of strength and particle size distribution, manganese slag is not suitable to be used as subgrade filler directly, and it needs to be treated by solidification. In this study, quicklime was used to solidify the manganese slag. For the solidified manganese slag, a compaction test and a *CBR* test were carried out to study its engineering characteristics.

#### 2.3.1. Compaction Test

**Figure 7:**
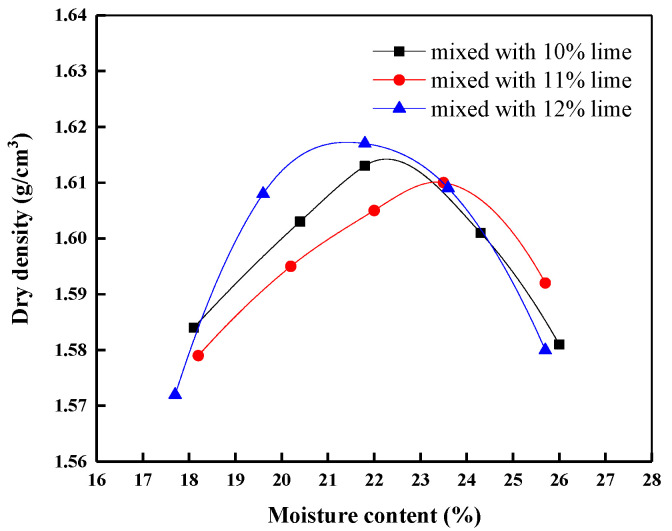
Compaction curve of lime-solidified manganese slag.

Mix 10%, 11%, and 12% lime into manganese slag, respectively to compact the mixture of lime and manganese slag. The compaction curve of manganese slag with different lime content is shown in Figure 7. With the gradual increase in lime content, the compaction curve gradually shifts to the upper right, indicating that the incorporation of lime increases the optimal moisture content of manganese slag and reduces the maximum dry density. The optimum moisture content of uncured manganese slag is 20.1%, and the corresponding maximum dry density is 1.71 g/cm^3^. When 10% lime is added, the optimum moisture content is 21.8%, and the maximum dry density is 1.613 g/cm^3^. With the increase in lime content to 11% and 12%, the optimal moisture content and maximum dry density change little, indicating that when the lime content reaches 10%, the continuous increase in lime content has limited improvement on the compaction effect of manganese slag.

Based on the above test results, it can be found that the incorporation of lime improves the compaction characteristics of manganese slag. When the moisture content of the uncured manganese slag is higher than the optimal moisture content of the subgrade filler in the project, it is generally necessary to carry out drying treatment to control the moisture content near the optimal moisture content. The addition of lime improves the optimal moisture content of the manganese slag, reduces the gap between the moisture content of the uncured manganese slag and the optimal moisture content, reduces the drying time, shortens the construction period, and saves the construction cost.

#### 2.3.2. *CBR* Test

**Figure 8:**
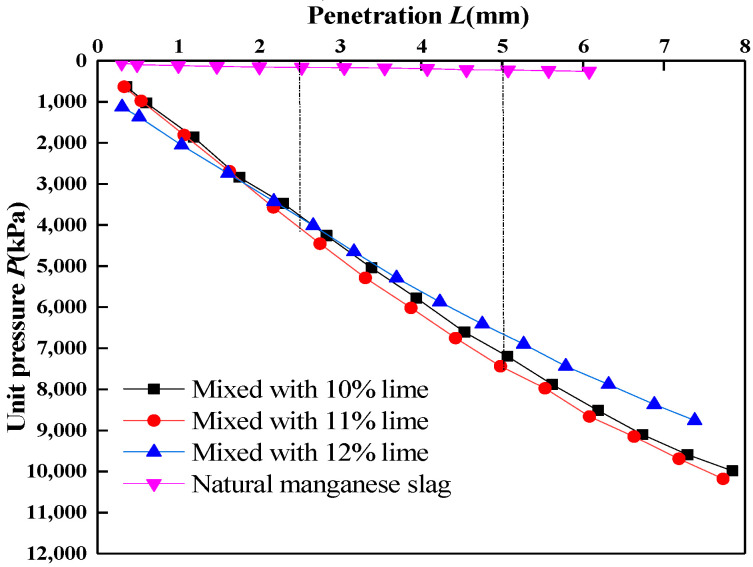
The relationship curve between unit pressure and penetration of lime-solidified manganese slag.

A total of 10%, 11% and 12% lime were added into the manganese slag, and the *CBR* test was carried out after the lime manganese slag mixture was compacted. The relationship between the penetration amount and the unit pressure under different lime content is drawn as shown in Figure 8. The results were compared with the *CBR* curves of uncured manganese slag. It can be seen from Figure 8 that with the addition of lime, the required unit pressure increases obviously under the same penetration, which indicates that lime solidifies the bearing capacity of manganese slag. According to the relationship curve between penetration and unit pressure, the *CBR* value of manganese slag with different lime content is calculated (Table 6).

The *CBR* test results (Figure 8) showed that the *CBR* value of uncured manganese slag is relatively low. When the penetration is 2.5 mm, *CBR*_2.5_ = 2.3, and when the penetration is 5.0 mm, *CBR*_5.0_ = 2.2, which does not meet the minimum *CBR* standard of subgrade. When 10% lime is mixed, the value of *CBR*_2.5_ increases to 55, with an increase of 27.5 times; the value of *CBR*_5.0_ increased to 68, an increase of 30 times. With the increase in lime content to 11% and 12%, the *CBR* value of manganese slag does not increase obviously. When the lime content is 12%, the *CBR* value even decreases slightly. Therefore, the addition of 10% lime produced a significant solidification effect on the *CBR* value of manganese slag, and its *CBR* value fully reached the standard of subgrade design.

According to the compaction curve of lime-solidified manganese slag in Figure 7, the optimum water content corresponding to the lime content of 10%, 11%, and 12% is 21.8%, 23.5%, and 21.7%, respectively. According to the *CBR* values of manganese slag under different lime content in Table 7, when the lime content is 10%, 11% and 12%, the *CBR* values are 68%, 73.5% and 64%, respectively, which meet the subgrade design standards. Therefore, 10% quicklime content is selected as the best mix proportion of solidified manganese slag.

## 3. Numerical Simulation

### 3.1. Methods and Theory

#### 3.1.1. Theory of Strength and Slope Stability

Fredlund’s double stress variable formula is used as the unsaturated shear strength theory:

(5) $$s=c^{\prime }+\sigma_{n}tan\varphi^{\prime }+\left(u_{a}-u_{w}\right)tan\varphi^{b}$$

where $c^{\prime }$, $\varphi^{\prime }$ are effective strength parameters;$\;\sigma_{n}$ is net normal stress, the difference between normal normal stress and pore gas pressure; $u_{a}$ is the pore gas pressure;$\;u_{w}$ is the pore water pressure; and $\;\varphi^{b}$ is angle indicating the rate of increase in shear strength with respect to a change in matric suction. The stability analysis of the slope adopts the Morgenstern–Price method in the limit equilibrium method. The Morgenstern–Price method can meet the balance of forces and moments in all directions, and can solve the sliding arc surface with arbitrary shape. The assumption of this method is that there is no tension between the soil strips, the ratio of the tangential force at the bottom of the soil strip to the horizontal thrust is the product of the undetermined parameter λ and the inter-strip force function f(x). The safety factor of slope stability is obtained by solving the differential equations of force and moment balance. 

#### 3.1.2. Monte Carlo Theory

The single method of using the safety factor to represent the safety state of the slope cannot reflect the risk state of the slope. Due to the spatial variability of soil, the randomness of soil parameter distribution also needs to be considered in the actual finite element calculation. The Monte Carlo method [23] is a numerical method based on a large number of random event statistics to obtain event probability characteristics such as expectation or probability distribution, which are connected with the solution of mathematical analysis, and use experimental methods to solve approximate solutions to problems such as mathematics, physics and engineering technology by experimental method.

In this study, random sampling was performed on the bulk density, cohesive force, and internal friction angle of the slope soil parameters. The number of times Fn < 1 is counted as *M*, which is recorded as failure times. The total sampling times is counted as *N*, and the failure probability can be expressed as:

(6) $$P_{f}=P\left(P_{n}\leq 1\right)=\frac{M}{N}$$

the mean value can be expressed as:

(7) $$\mu_{F}=\frac{1}{N}\overset{N}{\underset{j=1}{\sum }}F_{j}$$

the standard deviation can be expressed as:

(8) $$\sigma_{F}={\left[\frac{1}{N-1}\overset{N}{\underset{j=1}{\sum }}{\left(F_{j}-\mu_{f}\right)}^{2}\right]}^{\frac{1}{2}}$$

the critical safety factor of landslide is defined as $\mu^{\prime }$, and the reliability index can be defined as:

(9) $$\beta =\frac{\mu_{F}-\mu^{\prime }}{\sigma_{F}}$$

the failure probability can be defined as:

(10) $$P_{f}=1-\Phi \left(\beta \right)$$

### 3.2. Finite Element Simulation of Slope Stability

#### 3.2.1. Finite Element Model

**Figure 9:**
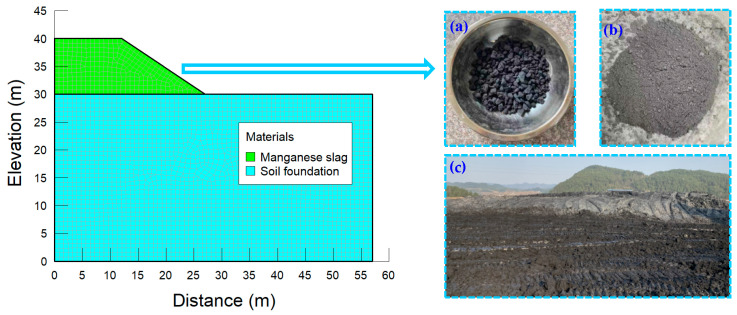
Finite element model and field material.

In this study, the finite element simulation of the stability of a highway slope was carried out by using the GeoStudio software. Figure 9 shows the finite element model of the slope, the slope height of 10m and the slope ratio of 1:1.5. It is assumed that the failure of the manganese slag highway slope obeys the Mohr Coulomb criterion. The model mesh is divided into 3113 nodes and 3008 elements.

#### 3.2.2. Calculation Parameters and Working Conditions

**Figure 10:**
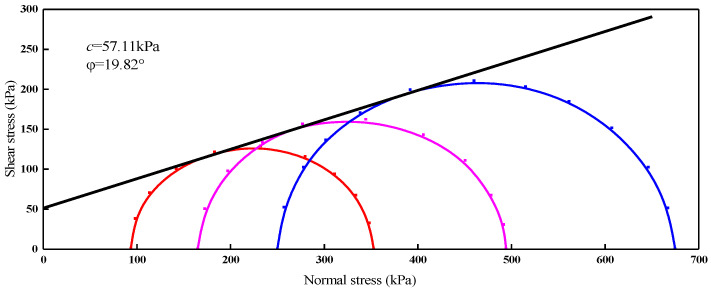
Envelope of consolidated undrained shear strength.

The cohesion and friction angle of manganese slag is determined by the triaxial shear test, it should be noted that manganese slag doped with 10% lime is used for the triaxial test (Figure 10). Salazar et al. presented a new method to measure the volume and volumetric strains of soil specimens during the triaxial test [24]. Mehdizadeh et al. developed a modified triaxial apparatus connected to a water supply system and collection tank to investigate the post-erosion behavior of soil under different loading patterns in undrained conditions [25]. In this study, the data of numerical simulation are from experiments [26]. The manganese slag in this state was solidified in advance. The calculation parameters (Table 7 and Table 8) of the finite element model were measured through the field test. 

**Figure 11:**
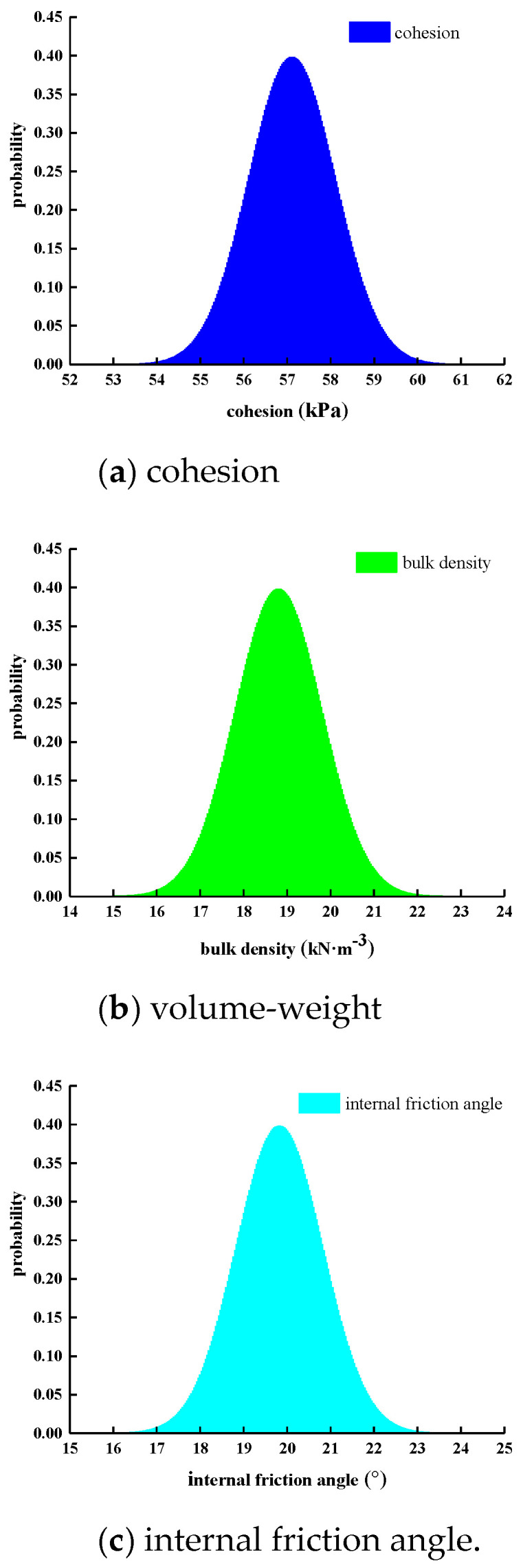
Probability density function distribution of manganese slag parameters.

**Figure 12:**
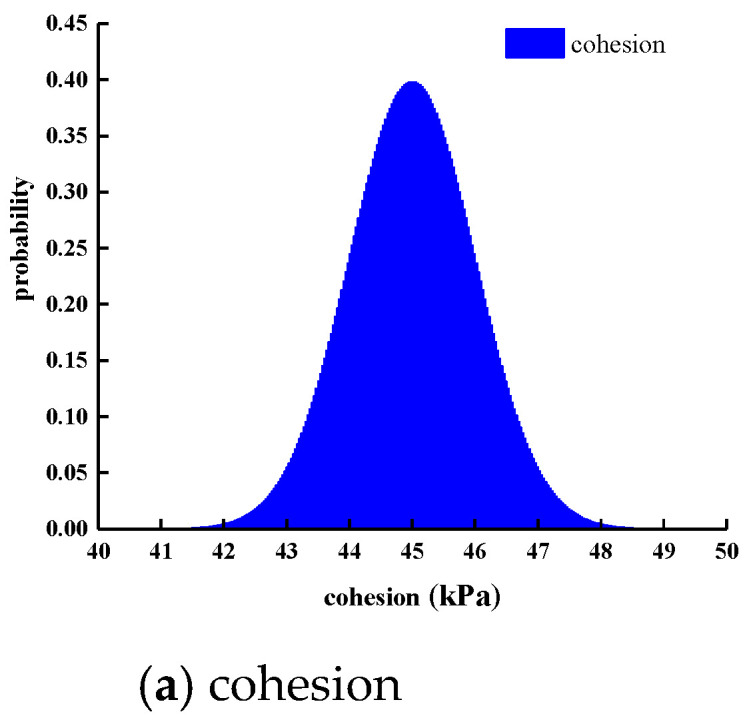

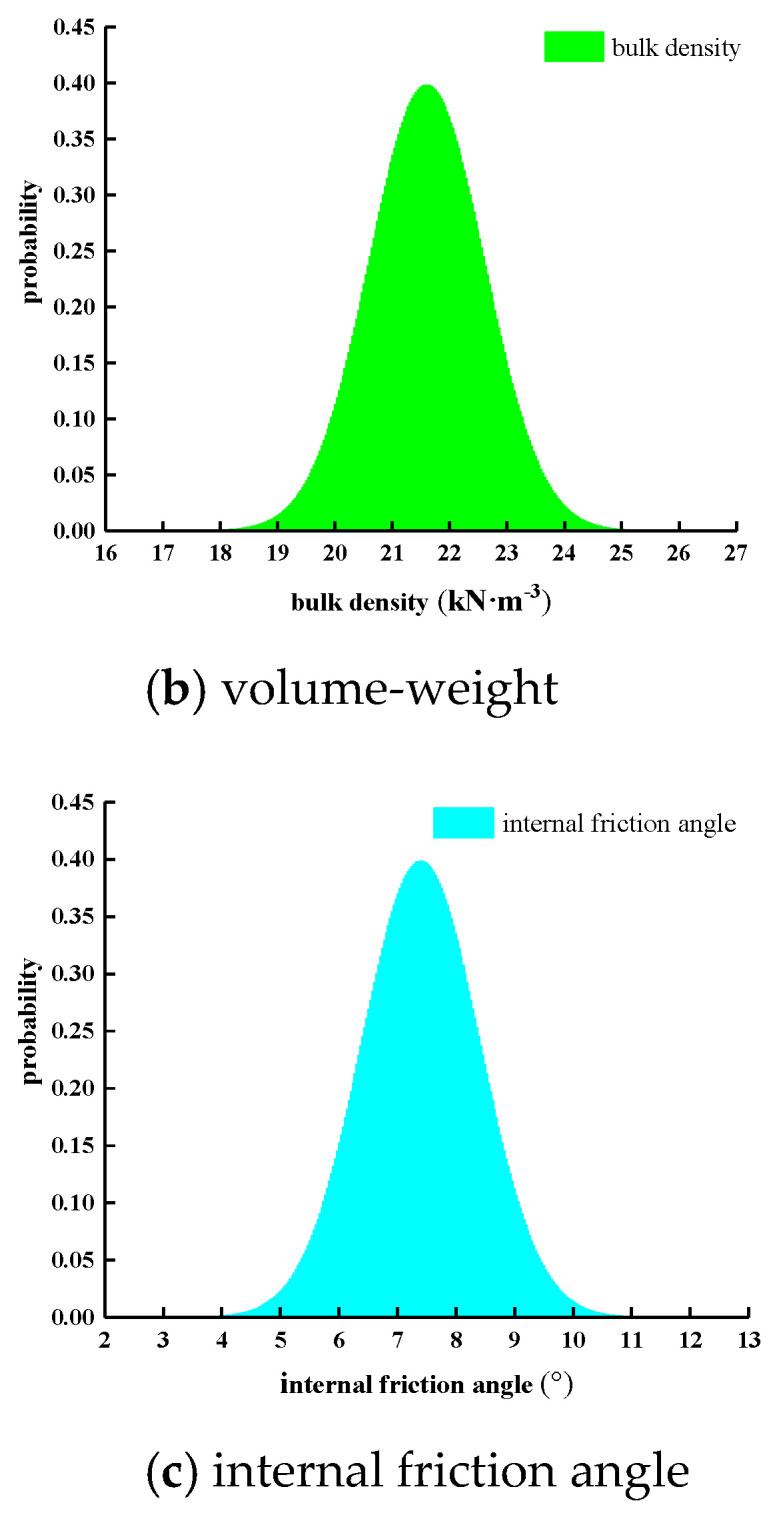
Probability density function distribution of clay layer.

Considering the influence of continuous rainfall, the rainfall intensity in the finite element model is respectively taken as 0 mm/d, 10 mm/d, 30 mm/d, and 50 mm/d. The rainfall duration in the model was set to 10 days, and the total calculation time was set to 30 days. The number of calculation steps was set to 30 steps. Considering the spatial variation in the parameters of the manganese slag slope material, it was assumed that the material parameter distribution obeys the normal distribution, the standard deviation of the parameters was set to 1, the number of Monte Carlo sampling was 2000. The normal probability density functions of the physical and mechanical parameters (cohesion, volume-weight, internal friction angle) of manganese slag and clay layer are shown in Figure 11 and Figure 12.

#### 3.2.3. Reliability and Failure Probability Analysis 

The stability of the manganese slag slope was simulated by Monte Carlo random sampling for 2000 times, and the reliability index and failure probability of the manganese slag slope under different rainfall intensities were calculated. In this study, the reliability index and failure probability are taken as the main indexes to evaluate slope stability. At the same time, the average safety factor, reliability index, minimum safety factor, maximum safety factor, and other index parameters under various working conditions are also calculated.

**Figure 13:**
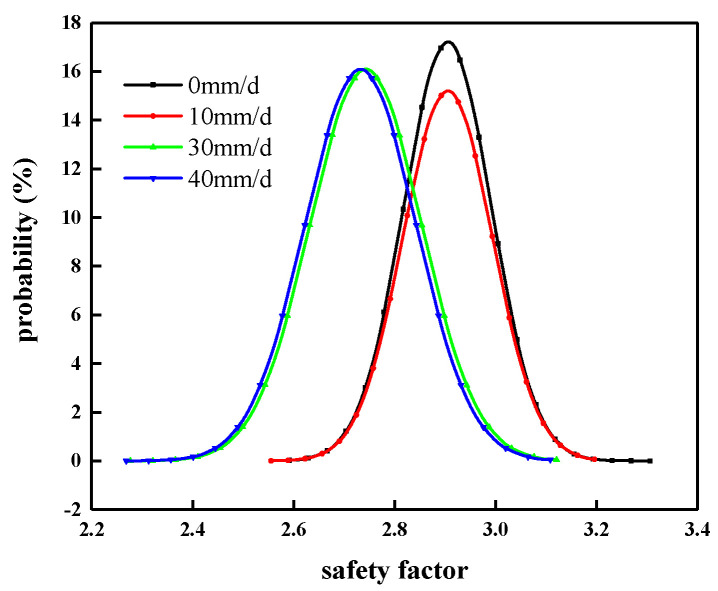
Probability distribution of slope safety factor.

**Figure 14:**
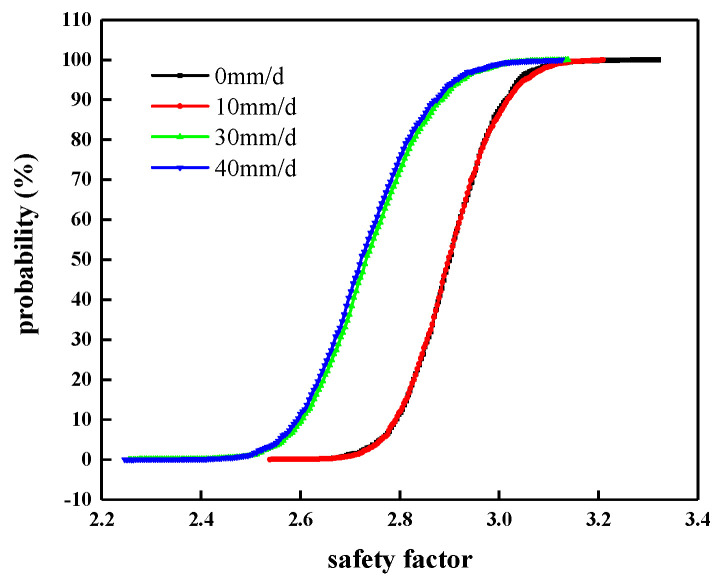
Probability density distribution of slope safety factor.

The probability and probability density distribution of the slope safety factor under different rainfall intensities are shown in Figure 13 and Figure 14, respectively. The statistics of various indicators are shown in Table 9.

In combination with Figure 13 and Figure 14, and Table 9, it can be seen that under all rainfall intensity conditions, the failure probability of the manganese slag slope is 0, which means that the manganese slag slope is in a safe state at this time. When the rainfall intensity is 0 mm/d, the average safety factor is 2.905, the reliability index is 21.778, the minimum safety factor is 2.573, and the maximum safety factor is 3.323.

When there is no rainfall, the failure probability of the manganese slag slope is less than the working condition when rainfall occurs. With the increase in rainfall intensity, the average safety factor, reliability index, minimum safety factor, and maximum safety factor of manganese slag slope under various conditions generally tend to decrease, which indicates that rainfall reduces the safety factor, and the greater the rainfall intensity, the greater the reduction in rainfall on the safety factor, the lower the reliability index. When the rainfall intensity is 50 mm/d, the average safety factor is 2.733, the reliability index is 15.762, the minimum safety factor is 2.247, and the maximum safety factor is 3.130. The failure probability under different rainfall intensities is 0. This is because the manganese slag slope safety factor is greater than one during the random sampling process, which is in a safe state. It also shows that although the manganese slag material is a solid waste slag, its water resistance is good, its stability meets the design requirements, and it can replace earth and stone materials to build highway roadbeds.

## 4. Conclusions

In this study, the physical and chemical characteristics of manganese slag were analyzed based on X-ray diffraction, X-ray fluorescence spectrum, SEM scanning and a particle analysis test. At the same time, the engineering characteristics of manganese slag before solidification and after solidification with quicklime were analyzed through heavy a compaction test and a *CBR* test. Then, the stability of a highway slope was simulated by the finite element method based on the Monte Carlo method. The conclusions are as follows:

(1) The XRD and XRF test results of manganese slag show that the crystallinity of quartz and hemihydrate gypsum (CaSO_4_-(H_2_O)_0.5_) in manganese slag is high, the content of silicon dioxide and silicon trioxide is high, and some metal oxides are also contained. The crystal structure and material composition of manganese slag make it appear as fine particles when the moisture content is low, and become slurry after being eroded by rain;
(2) The SEM results of manganese slag show that the size distribution of manganese slag particles is uneven, there are a lot of pores between the particles, and there is no obvious cementation between the particles, so the solidification of manganese slag should be considered in practical application;
(3) The engineering characteristic test of manganese slag shows that the particle size distribution characteristics and critical moisture content of manganese slag are similar to those of silt. The content of sand and powder particles is relatively high, and the content of clay particles is relatively small. It belongs to high plasticity silt in the plastic map. The optimum moisture content of manganese slag is 20.1%, the corresponding maximum dry density is 1.71 g/cm^3^, and its *CBR* value is about 1.5~2.3. It does not meet the subgrade design standard, and the manganese slag in an uncured state cannot be directly filled as subgrade filler;
(4) Mixing with lime can improve the compaction characteristics of the manganese slag, increase the optimal moisture content of the manganese slag, and reduce the gap between the moisture content of the unsolidified manganese slag and the optimal moisture content. Mixing with 10% lime can produce a significant solidification effect on the *CBR* value of manganese slag, and its *CBR* value can fully reached the standard for subgrade filling;
(5) The greater the rainfall intensity, the lower the safety factor of manganese slag slope, and the lower the slope reliability index. In the actual project operation and management process, the stability of the manganese slag slope under high-intensity rainfall conditions should be paid attention to. Considering the spatial variability of manganese slag and clay layer, the failure probability of manganese slag slope is 0 under different rainfall intensities conditions.

## Figures and Tables

**Table 1:** Main technical specifications of X-ray photoelectron spectrometer.

Vacuum Degree of Analysis Chamber	Optimal Energy Resolution	Minimum Spatial Resolution	Electronic Counting Rate	Sensitivity
5 × 10^−10^ mbar	0.43 eV	1 μm	4 Mcps	1,000,000

**Table 2:** Element content of manganese slag.

Element	O	Si	S	Al	Ca	Fe	Mn	Mg	K	Total
Percentage (%)	47.77	16.38	10.36	6.62	5.17	3.83	3.56	2.38	1.99	98.06

**Table 3:** Chemical composition of manganese slag.

Chemical Composition	SiO_2_	SiO_3_	CaO	Al_2_O_3_	Fe_2_O_3_	MnO	MgO	K_2_O	Total
Percentage (%)	35.04	25.88	7.23	12.50	5.48	4.59	3.94	2.93	97.59

**Table 4:** Analysis of particle composition of manganese slag.

Sample Name	Percentage of Each Particle Size (mm) in Total Mass (%)		
Manganese Slag	>0.02 (Sand)	0.02~0.002 (Powder)	<0.002 (Cosmid)
	32.43	60.20	7.37

**Table 5:** Calculation results of *CBR* value.

Sample Number	Penetration L/mm	Unit Pressure P/kPa	*CBR* Value/%	Water Absorption/g	Expansion Rate/%
sample 1	2.5	110	1.6	238	6.46
	5.0	161	1.5		
sample 2	2.5	132	1.9	224	6.37
	5.0	198	1.9		
sample 3	2.5	161	2.3	203	6.13
	5.0	227	2.2		

**Table 6:** *CBR* value of manganese slag with different lime content.

Material	Penetration/mm	Unit Pressure/kPa	*CBR* Value/%
Mixed with 10% lime	2.5	3850	55
	5.0	7140	68
Mixed with 11% lime	2.5	4095	58.5
	5.0	7717.5	73.5
Mixed with 12% lime	2.5	3850	55
	5.0	6720	64
Natural manganese slag	2.5	161	2.3
	5.0	227	2.2

**Table 7:** Parameters of seepage calculation.

Partition	Volume-Weight (kN/m^3^)	Permeability Coefficient (m/s)	Saturated Volumetric Moisture Content (m^3^/m^3^)
Manganese slag	18.8	1.38 × 10^−7^	0.417
clay layer	21.6	5.21 × 10^−8^	0.15

**Table 8:** Parameters of stability calculation.

Partition	Poisson’s Ratio	Internal Friction Angle (°)	Cohesion (kPa)	Elastic Modulus (MPa)
Manganese slag	0.25	19.82	57.11	174.6
clay layer	0.35	7.4	45	35

**Table 9:** Statistics of various indexes of manganese slag slope under different rainfall intensities.

Rainfall Intensity	0 mm/d	10 mm/d	30 mm/d	50 mm/d
Index				
Average safety factor	2.905	2.905	2.743	2.733
Reliability index	21.778	21.477	15.787	15.762
Probability of failure (%)	0	0	0	0
Minimum safety factor	2.573	2.539	2.255	2.247
Maximum safety factor	3.323	3.213	3.142	3.130

## Data Availability

The data presented in this study are available on request from the corresponding author. The data are not publicly available due to the project restrictions.
